# Sources of stress among Federal Correctional Officers in Canada

**DOI:** 10.1177/00938548231174900

**Published:** 2023-05-29

**Authors:** Marcella Siqueira Cassiano, Rosemary Ricciardelli

**Affiliations:** Memorial University

**Keywords:** correctional office, correctional work, stress triggers, staff, co-worker, manager, prison resident

## Abstract

Most correctional officers describe their jobs as stressful. The current study advances the scholarship on correctional stress by offering a rare qualitative analysis that identifies, provides meaning, and contextualizes sources of stress in correctional services. This study complements the correctional stress literature, which, until now, has relied primarily on quantitative methodologies to identify and assess stress determinants. Forty-four correctional officers from Canada’s federal prisons were interviewed about their primary source of stress. Findings indicate that staff (i.e., co-workers and managers), not prison residents, represent a primary source of stress in correctional work. In addition, job seniority and gossip were the main stress triggers associated with co-workers, while centralization of decision-making processes and a lack of instrumental communication and support triggered stress coming from managers.

## Introduction

Most correctional officers (henceforth “COs”) describe their jobs as stressful. In addition to enforcing rules and maintaining routines at correctional institutions, COs experience continued exposure to suffering, harm, and violence. Their job also entails witnessing or responding to critical incidents, such as psychological distress, self-harm, stabbings, and suicide, while also managing the risk of being victimized by prison residents (henceforth “residents”; Ellison & Jaegers, 2022). In addition, COs navigate staff-perpetrated harms and an often “toxic” workplace culture (Burdett et al., 2018). The risks and stressors underpinning correctional work may result in the officer developing mental health injuries such as generalized anxiety disorder and posttraumatic stress disorder (Regehr et al., 2021). Concerned with the occupational realities of correctional work and how it affects workers’ health and well-being, scholars have dedicated considerable effort to measuring stress levels and identifying its determinants. This article discusses sources of stress in Canada’s federal correctional system based on interviews with 44 COs. The data supporting this article was collected under CCWORK (Ricciardelli et al., 2021), a multiyear mixed-method longitudinal study of CO’s occupational well-being.

## Occupational Stress in Correctional Work

The literature on stress in correctional work is vast, primarily quantitative, and mostly based on studies conducted in the United States. Job stress, often associated with job satisfaction and organizational commitment, is the leading topic of correctional research (Butler et al., 2019). Scholars use correlations and regression analyses to order stress determinants according to their impact on well-being. A careful examination of the literature reveals three somewhat intertwined types of stress determinants in correctional work (Lambert et al., 2006): job stressors (i.e., intrinsic to the job); organizational stressors (i.e., resulting from the organizational structure); and employee characteristics, which are usually addressed as a potential correlate of job and organizational stressors.

### Job Stressors

Job stressors include how COs perceive their job (i.e., as safe or dangerous; Cullen et al., 1985), their professional worthiness (Shamir & Drory, 1981), and work arrangement (e.g., workload, overtime, and understaffing; Finney et al., 2013). Despite covering many topics, the job stressor literature within correctional services has emphasized two themes: work arrangement, particularly workload, and role problems. These two themes deserve attention as they are relevant for the current article.

Intuitively, more work would mean more stress and vice versa. However, studies on work arrangement have presented inconsistent findings. Although several studies have demonstrated that increased workload was a predictor of stress (Dignam & West, 1988; Huckabee, 1992; Schaufeli & Peeters, 2000; Schiff & Leip, 2019), others (Castle, 2008; Moon & Maxwell, 2004b) have found that over time, which technically presupposes an excessive workload, was not a stress determinant. Financial gain might mitigate any stress caused by voluntary overtime. Researchers studying work arrangements have also explored and confirmed boredom, a common feature of correctional work, as a source of stress (Hughes & Zamble, 1993; Hughes, 1991).

The scholarship on role conflict and stress emerged in the 1980s with correctional philosophies that required correctional services to reconcile custody with the care and rehabilitation of federally sentenced men and women. The need to reconcile potentially conflicting demands has made numerous COs feel frustrated and burnt out due to role conflict (Arnold, 2005, 2016; Crawley & Crawley, 2008; Cullen et al., 1985; Liebling et al., 2011; Philliber, 1987). To explain the frustration and the stress stemming from changes in the correctional role and mind-set, scholars have explored whether COs’ orientations (i.e., punitive vs rehabilitative) toward correctional work influenced stress levels. Scholars have demonstrated that COs with a punitive orientation experienced higher stress levels (Cheeseman et al., 2011; Cullen et al., 1985; Dignam et al., 1986; Ferdik & Hills, 2018; Misis et al., 2013; Toch & Klofas, 1982; Whitehead, 1989). As prison reforms advanced, COs’ orientations became multidimensional and nuanced, and dualist orientation analyses became outdated. However, recent studies found that COs’ views of residents were still significant in determining employee well-being. For instance, studies in the United States have demonstrated that officers who perceive interactions with residents as “dangerous” are more likely to present higher stress levels than others (Lambert & Paoline, 2008; Misis et al., 2013). Viotti (2016), in one of the few qualitative accounts on CO stress available, has found that every participant in her sample of 28 Italian COs reported feeling overwhelmed by residents’ requests, which were numerous and urgent amid short staffing. COs also expressed difficulties managing residents’ emotional reactions and aggressive behaviors.

COs, to reconcile their role as providers of care, control, and custody, began to seek standardized procedures for interacting with residents (Toch & Klofas, 1982). However, in most cases, such procedures were unavailable. Scholars have analyzed the lack of standardized procedures as a form of “role ambiguity” (i.e., uncertainty about which tasks and responsibilities), exploring its positive correlation with stress (Lasky et al., 1986; Stohr et al., 1994). However, role ambiguity seems to have lost part of its significance as a research domain as correctional work became more specialized through improving training. At least, role ambiguity transitioned from a job stressor to an organizational stressor (Liebling et al., 2011), often associated with the quality of training, management, and communication, as well as autonomy to make decisions (Lambert et al., 2006).

### Organizational Stressors

The literature on organizational stressors often discusses the centralization of decision-making, instrumental communication, organizational justice, leadership quality, and social support (i.e., perceptions of being cared for). Leadership quality includes the manager’s ability to support and communicate with team members. Research led by Lambert on the United States reality has demonstrated that centralization (i.e., lack of participation in decision-making) and unclear communication were associated with stress (Lambert et al., 2006). Lambert’s studies echo research from the 1980s and 1990s (Cheek & Miller, 1983; Lindquist & Whitehead, 1986; Shamir & Drory, 1981; Wright et al., 1997). Expanding the analysis to broader correctional policies, Jurik and Winn (1987) have demonstrated that the inability to influence institutional policy decisions also stresses COs.

Addressing organizational justice, Lambert and colleagues have demonstrated that COs who perceive correctional processes and outcomes as fair (i.e., procedural justice) and legit (i.e., distributive justice) tended to present lower stress levels (Lambert et al., 2006; Lambert et al., 2007). More recently, Lambert and colleagues have explored COs’ interpersonal justice perceptions, demonstrating that COs who felt respected by supervisors and administrators presented lower stress levels (Lambert et al., 2019). Meanwhile, leadership quality, another organizational stressor, remains an issue among COs in the United States, Canada, and elsewhere (Schaufeli & Peeters, 2000). Ineffective leadership, including overly strict supervision (Lindquist & Whitehead, 1986), inconsistent instructions (i.e., a lack of instrumental communication; (Breen, 1986; Cheek & Miller, 1983; Lindquist & Whitehead, 1986), and unsupportive management (Hughes & Zamble, 1993; Hughes, 1991; Lindquist & Whitehead, 1986; Peeters & Buunk, 1995), are stress-inducing.

Management deserves special attention, working as both a stressor and a coping (i.e., protective) mechanism within correctional work. Studies in Canada, England, the Netherlands, and the United States revealed that unsupportive management could lead COs to feel inferior (Dilulio, 1987; Peeters & Buunk, 1995), stressed (Dollard & Winefield, 1995; Hughes & Zamble, 1993; Hughes, 1991), and burnt out (Shamir & Drory, 1981). Meanwhile, support can help COs cope with occupational stressors (Cheek & Miller, 1983; Dignam & West, 1988; Misis et al., 2013). Misis and colleagues (2013) have shown the protective effect of management support on CO stress was more significant than the risk effect (i.e., potential threat) posed by residents.

The stress generated by the unsupportive management can affect the CO’s job performance and eventually affect correctional workers’ and residents’ safety and security. Leaving COs feeling uncared for by managers and institutions can have disastrous consequences for the correctional system, especially an adversarial relationship between COs and residents. Officers feeling uncared tend to compare themselves to residents in terms of dispensed care and assistance and question organizational justice (Toch & Klofas, 1982), when both population categories should be treated with care and respect. Furthermore, comparing COs and residents can and often does fuel discourses and practices that hierarchize human worthiness based on moral criteria, generating an “us versus them” divisiveness within correctional services that can affect employee morale and undermine the CO-resident relationship and, ultimately, the resident’s well-being. However, management support alone is insufficient for maintaining a healthy work environment in correctional institutions; peer support, including loyalty and collegiality, also matters.

Peer support can make-or-break correctional work, and training and work arrangement help explain the significance of peer support for COs. First, COs usually work in pairs for safety reasons. Second, correctional training guides recruits to perceive co-worker dependability (i.e., reliability) as an essential attribute of a “good” officer (Cassiano et al., 2022). Thus, friendly interactions with co-workers improve job satisfaction, while unfriendly interactions foster a toxic work environment. Finally, although scarce, the scholarship on peer support (Schaufeli & Peeters, 2000) has confirmed that peer support can increase (Grossi & Berg, 1991; Morrison et al., 1992; Peeters & Buunk, 1995) or reduce stress levels (Cullen et al., 1990; Dollard & Winefield, 1995; Van Voorhis et al., 1991), depending on the relationship’s configuration. For instance, a recent study conducted with 1,083 COs from the United States found that staff support (i.e., supervisor, co-worker, and administrative support) is a “buffer” against correctional work stress (Walters, 2022). This study also found that weak staff support is more stressful for COs than dealing with residents. The information that staff can be a source of stress in correctional services is not a novelty.

A Canadian study from the late 1980s (Breen, 1986) has concluded that negative working relationships with co-workers were the second most remarkable source of stress among COs after “bureaucratic decision-making” (including unfair promotions), as noted by 90% of the study’s 117 participants. Another Canadian study (Hughes & Zamble, 1993) has reported co-workers as the second most significant, despite being the most cited, source of stress for correctional. The other sources of stress were unsupportive managers, boredom, and residents (Hughes & Zamble, 1993). A few studies from the United States have reported similar results, listing management, co-workers, and residents, in descending order, as primary sources of stress in correctional work (Cullen et al., 1985; Owen, 1988; Tewksbury & Higgins, 2006).

Individual characteristics (e.g., gender, age, race, education, tenure, and institutional security level) and work–life balance also affect CO stress levels. However, research on these factors has reached equivocal or null results (Butler et al., 2019; Lambert et al., 2007; Schaufeli & Peeters, 2000; Walters, 2022); stress and these factors, except for work–life balance, are often not marked by a linear relationship (Dowden & Tellier, 2004).

The studies reviewed in this section, except for Viotti’s research with Italian COs, miss the opportunity to explore the intricacies and nuances of COs’ perceptions of stress due to their quantitative nature. Despite their high quality and usefulness in identifying and hierarchizing stress sources, these studies have asked COs to rank stressors in a list of pre-selected items that may not necessarily reflect the participant’s source of stress. Moreover, the list may include items deemed stressful compared with peer items but not too stressful in the grand scheme of correctional work. In addition, although effective in producing generalizable conclusions, quantitative research provides limited contextual information on the reasons underpinning each source of stress.

## Current Study

This study identified and analyzed sources of stress among COs from 12 federal prisons in Canada. Stress is a physical and emotional reaction to job demands and workplace adversities (Cullen et al., 1985). This article advances the scholarship on COs’ occupational stress—which to date has focused primarily on identifying stress determinants quantitatively—by offering a qualitative analysis of the topic, which explores the reasons underpinning stress. Forty-four COs were asked about their primary source of stress and were allowed to explain the reasons and context underlying their stress.

## Method

### Project and Data

The 44 interviews used to support this article derived from the qualitative component of CCWORK (Ricciardelli et al., 2021). This ongoing (2018-2028) mixed-method longitudinal study explores job and organizational contexts to explain the high prevalence of occupational stress injuries among federal COs in Canada. Such injuries include major depressive disorder, anxiety disorders, posttraumatic stress disorders, and suicide ideation (Carleton et al., 2019; Carleton et al., 2021). CCWORK was designed based on the assumption that work conditions and outcomes, including prison workers’ health and wellness, are intertwined with conditions of incarceration (Ricciardelli & Power, 2020). Thus, the knowledge that informs improvements in work conditions and employee health creates opportunities to improve the quality of correctional services provided to residents. CCWORK participants work at federal correctional facilities housing about 12,300 men and women sentenced to 2 or more years in custody (Correctional Service Canada [CSC], 2022a), which are administrated by Correctional Service Canada (CSC).

As a longitudinal endeavor, CCWORK collects clinical, quantitative, and qualitative data from COs at the end of their correctional training program (baseline) and annually after that (follow-up waves). All CO recruits, about 500 individuals are targeted based on the second author’s correspondence with CSC. However, not everyone enrolls in the project, and not every participant completes every component. For example, the enrollment rate in the qualitative part has varied between 23% and 25% (115-125 individuals) in the first 3 years of CCWORK. CSC facilitates CCWORK by advertising the project to CO recruits and allowing interviews during paid work time in private space, where the project team (CCWORK’s investigators, postdoctoral research fellows, and graduate students), including the authors, conduct interviews in person or, since the pandemic, over the phone. Interviews last 45 to 90 min and are voice recorded and transcribed verbatim. Despite CSC’s collaboration, participation in CCWORK is voluntary, and no monetary incentives are provided. Despite collaborating with the project and facilitating data collection, CSC has no access to the research data, which is anonymized during the analysis. Participants are assigned an identification number at project enrollment to facilitate longitudinal tracking. CCWORK interviews are semi-structured. They inquire into the officers’ expectations, experiences, and perceptions of correctional work, including stressors—the topic of this article.

To capture participants’ experiences and perceptions of stress, the interview team first asked participants the following question: “Where do you think most of your stresses come from?” Then, if necessary, the team probed about whether stress came from residents, colleagues, management, or the public (e.g., media coverage). Probing topics were determined based on findings from the correctional stress literature produced in Canada (Breen, 1986; Hughes & Zamble, 1993) and beyond (Cullen et al., 1985; Liebling et al., 2011; Owen, 1988; Tewksbury & Higgins, 2006; Walters, 2022). In addition, when participants indicated staff (i.e., colleagues or managers) as their primary source of stress, the interview team followed up by asking why participants did not consider residents stressful. This line of inquiry had two objectives: First, challenge portrayals of the CO-resident relationship as antagonistic and stressful (Liebling, 2000); Second, encourage comparisons between different stressors, which could reveal further information on stress dynamics. The research ethics protocols adopted received approval from Memorial University’s ethics board (File No. 20190481).

### Sample

The 44 interviews used in this article represent a sub-sample (i.e., a snapshot) of 72 follow-up interviews (Wave 1) conducted with COs who joined the project’s qualitative component in 2018/2019 (i.e., Year 1 cohort). This cohort comprised 118 participants at baseline, dropping to 72 individuals in follow-up one (attrition from baseline to Wave 1 in the Year-1 cohort was about 40%). At baseline, the 118-individual cohort included COs recruits going to CSC’s 43 correctional institutions in all 5 regions (i.e., Atlantic, Quebec, Ontario, Prairie, and Pacific). The sub-sample was drawn based on convenience, as those 44 interviews had been transcribed and coded and were ready for analysis. Thus, due to sampling and attribution between data points (i.e., baseline and Wave 1), the sub-sample used in this article does not include all prisons and regions.

### Analysis

Interview data were analyzed by the first author and a team of graduate research assistants following a four-step process. First, research assistants read the interview transcripts to identify answers to the question, “where do you think most of your stresses come from?” Using the software NVivo and a codebook (i.e., node tree) designed to organize all CCWORK interviews, research assistants coded the stress answers under a node labeled stressors in the occupational challenges, hazards, and stressors session of the master codebook. The coding process involved creating child nodes under the node stressors to reflect the participants’ primary source of stress. These child nodes were co-workers; management; prisoners (i.e., residents); other (participants saying that “everything” about correctional work was stressful); and without stress (for participants who reported having no stress from work). A few participants named two primary sources of stress. Those participants had their excerpts coded under the source emphasized during the interview. Two interrater reliability tests (Cohen’s Kappa) were performed during Step 1; results averaged 0.9884.

Second, the first author recoded the data under the nodes co-workers, management, and prisoners, creating child nodes reflecting participants’ stress rationale. Answers coded under other (two individuals) and without stress (one individual) were disregarded in this second step; they were too succinct, offering no contribution to this study. During recoding, the first author identified gossip and seniority as the primary triggers associated with co-worker stress. Management stress was associated with power centralization, communication issues, and unsupportive management (i.e., lack of managerial support). Stress coming from residents was associated with uncertainty and unpredictability, demands amid short staffing, role conflict, and a mismatch between job expectations and job reality. One participant provided two rationales to explain the same source of stress and was coded under two stress triggers. Seven participants did not provide much detail to explain their stress despite naming their source of stress. These participants were coded under the theme no details in Step 2, and their excerpts were not cited in the results section. Thematic saturation (Bowen, 2008) was achieved with 34 interviews; after the 34th interview, no more child nodes were created. Third, a senior research assistant reviewed Step 2 to ensure consistency. Fourth, IBM-SPSS was used to calculate the percentages for demographics, sources of stress, and stress triggers.

### Participants

Research participants (including those coded as other and without stress; *N* = 44) were from 12 of CSC’s 43 institutions. These institutions were in three of CSC’s administrative regions: Prairies (70.5%), Atlantic (22.7%), and Pacific (6.8%). About a third of participants worked in maximum security institutions (36.4%), while the remaining worked in the following types of institutions: in multilevel institutions with maximum, medium, and minimum-security units (27.3%); medium-security institutions (15.9%); minimum/medium-security institutions (13.6%); and correctional psychiatric centers (6.8%). Most participants (90.9%) were from men’s institutions, with 9.1% from women’s institutions (the sample included three of Canada’s six women’s institutions). About two thirds of participants self-identified as male (59.1%), while 40.9% self-identified as female. Participants identified as white (79.5%), Indigenous (11.4%), and racialized (4.5%). Over 80% of participants were aged 19 to 24 (29.5%) and 25 to 34 (54.5%), with the remaining aged 35 to 44 (9.1%), 45 to 54 (2.3%), and 55 to 64 (2.3%). Half of the participants (50.0%) were in a marital relationship (i.e., married or common-law relationship), while 43.2% had never married, and 4.5% were divorced. Most had a postsecondary degree: almost a third had obtained a university degree (27.3%) or a college diploma (52.3%); the others had a high-school diploma (18.2%) and a vocational degree (2.3%). Although 77.3% of participants had never worked in correctional services before, 22.7% had correctional experience before joining the federal prison system. One participant (2.3%) has not declared age and marital status, while 2 (4.5%) have not declared race. Participant demographics are somewhat consistent with Canada’s average federal CO population (Hughes & Zamble, 1993; Samak, 2003).

## Results

Participants (*N* = 44) reported co-workers (36.4%), residents (29.5%), and management (27.3%) as their main sources of stress. The remaining (6.8% or three participants) reported they either found everything about correctional work stressful (4.5%) or had no stress from work (2.3%). Staff (i.e., co-workers and managers) accounted for more than two thirds (63.7%) of the work stress experienced by participants. Co-worker stress was associated with gossip and seniority. Management stress was connected to power centralization, communication issues, and unsupportive management. Resident stress was linked to uncertainty and unpredictability, residents’ demands amid short staffing, and role conflict. All participants asked why they did not consider dealing with residents stressful (28 or 63.6%) associated their reasoning with a mismatch between job expectation and reality. This section details each source of stress and its respective stress triggers.

### Co-Workers

Participants who reported co-workers as their primary source of stress equated working in correctional services to navigating “drama,” “cattiness,” “rumor mills,” “negativity,” and having to prove their “worthiness” to deserve respect as an employee. Commenting on her sources of stress, P77 said: “I think it’s never the inmates; working with murders, working with rapists that’s easy. What bothers me is the staff.” P77’s answer deserves attention: despite having been “hit in the face” by a resident, P77 still viewed co-workers, not residents, as her primary source of stress. P87, like a third of the sample, said that co-workers were “the hardest part for sure.” Participants invoked two stress triggers related to co-workers: gossip, which was referred to as “drama” or the “rumor mill,” and seniority.

#### Gossip

For P149, “staff arguing” and “gossip” are “certainly a stressor” at her institution: “I don’t want to say it’s not bullying, but there is a lot of gossip that goes around; lots of backstabbing and talking behind people’s backs and stuff, and that’s certainly a stressor for me.” P105 portrayed gossip as a “culture” in his institution: “There is that culture here at this place of people talking behind your back. That’s a stressor for everybody that works here.” In contrast with seniority, an organizational stressor, gossiping seems associated with a job stressor—keeping vigilant, watching, and monitoring. Several participants portrayed sitting in a “static post” (i.e., the control room or “bubble”) as tedious. P90 said, without hesitation, that the “bubble” was his primary source of stress: “Yup, 100% yeah.” Officers dispelled boredom with small talk about themselves and others; gossip was used as a tool to endure correctional work. After acknowledging that gossip is “hard to deal with,” P105 attributed gossip to having a “boring job for 90% of the time”: “You have nothing to do but sit around and talk and bitch about people.” In the same fashion, P24 said that co-workers could get “super catty,” blaming idleness: “There’s a lot of downtime, so people just talk about things.” Echoing P105 and P24, P25 confirmed boredom as a stress trigger:

People might think maybe it’s the incidents, but we respond really well as a team here. It’s just the downtime and the different drama that I don’t personally like. I work a lot of twelve hours [shifts], so I’m in a lot of static posts. They have more time to gossip (P25).

P45 further explained how the control room work dynamics offer a fertile ground for gossip. Officers working in static posts tend to spend most of their shifts in the control room. They only leave to use the restroom and do their rounds (i.e., counting the resident population and verifying if everyone was well). Their work consists primarily of monitoring CCTV circuits and controlling the doors (i.e., access) within the prison. Thus, work can easily become boring.

You’re sitting there for such a long period of time. I’m fine with silence; I’m comfortable with silence, “we don’t need to talk, you can stay in your head, I’m staying in my head,” but some people are not comfortable with it, so they want to talk about themselves or other people or management or whatever, and I find that’s when I start to feel stress because I’m just in head: “Shut up!” (P45)

P45’s inability to tell her co-workers that she would rather work silently seems to be associated with a need to be accepted by co-workers, given how long they sit together in the same space. The impact of work arrangement in the control room on gossip became clear when P45 compared how she interacts with residents and co-workers: “[With residents], I keep all my conversations short, direct, straight to the point, but with your colleagues, you’re sitting there for such a long time.”

Feeding the gossip culture shielded officers from becoming a target in conversations and having their lives scrutinized by colleagues. For instance, P25 said: “[If] I’m not in the drama, I’m the center of the drama.” The only way to control information generated about themselves was to participate in the web of gossip marking the correctional work environment. Since participating in gossip was a form of self-protection, time off work was stressful for some, as it meant being talked about:

I work days for 2 weeks, and then I work midnights for 2 weeks and then I have a week off, I’m not here a whole lot. When I hear things, I’m like “oh cool that’s interesting! I didn’t know that I did that” [laughter] (P25).

P25 reported “a lot of anxiety” from gossip: “It’s hard. I get a lot of anxiety from it, but I never used to get anxiety.” Gossip worked as a mirror or a “looking-glass self” (Cooley, 1983) that officers used to gauge self-esteem and self-worth within workplace circles and interactions. Officers could not help but let their predictions of how others perceive them dictate their behavior and self-feelings. Thus, gossip that involved portraying co-workers as a “burden” or a “sack” was extremely stressful. Officers referred to as a “burden” questioned whether co-workers would have their back in the event of a critical incident, which undermined their perception of safety. Thus, officers dedicated considerable effort to managing the impression they believed they provoked in others to dissipate fear and stress. P33 described the stress of impression management that entails constantly watching and being watched:

If the line falls behind me or after me, then you need to make sure you’re not forgetting anything because, again, you don’t want to look like a sack. You don’t want to look like you don’t know what you’re doing. So, there are people looking to find that hole. You know?! They’re watching you to see if you’re going to mess up (P33).

Trying to do everything right in a context marked by insurmountable scrutiny and relentless gossip was not always enough to earn respect and dissipate any concerns of being rejected by co-workers. In fact, feeling that their work was in vain was a source of stress. P33 reported that her co-workers were not content, although she made sure everything was “right”:

OK, I want to make sure I did this right, this right, this right. That’s where I’ll have other people look at me and be like. “you need to slow down!” or “you need to relax a little bit.” And I’m like “fuck! There’s people who are watching right now.” They know I’m a hard worker and are not looking to point out faults. They are usually like: “it’s OK!” It doesn’t matter what they think. But that’s where my stressors come [from]. I don’t ever want to be viewed as somebody who’s not pulling their weight (P33).

Officers indicated that gossiping at work was also associated with feelings of isolation. Several participants reported distancing themselves from co-workers since they started with CSC because of staff “drama.” P27 “chose not to hang out with people outside of work.” However, distancing themselves from co-workers often made participants fear not being helped if a resident attacked them. P27 said that gossip eroded trust in co-workers, leading COs to feel isolated from co-workers and thus unsafe:

Most of my stress comes from the other officers; that feeling of isolation makes me question whether they will run to respond to you. That sort of thing definitely causes more stress than even walking through a crowd of 30 inmates knowing that you might get stabbed. It’s honestly higher stress (P27).

#### Seniority

Seniority, a system that rewards employees based on longevity, not merit, was viewed as a stress trigger for allowing senior officers to legitimately impose their work practices upon newer officers, regardless of if those practices complied with training, policies, and protocols. P77 described seniority as “control”: “People try to take control of a situation, and depending on your seniority, you’re just expected to follow. Right? Everything’s so seniority based. Seniority, seniority, seniority.” Despite indicating management as his primary source of stress, P31 reported receiving a lot of “pushbacks” from “older officers” for trying to apply what he learned in correctional training, which was stressful. P150 said that “doing everything right” was her primary source of stress because expectations at her institution were not uniform. Instead, they varied according to people and seniority. In the same fashion, P5 said that “getting everyone on the same page” was his primary source of stress.

P77 added that senior officers made hierarchy visible through questions that outed recently hired COs, such as “how long have you been here?” According to P77, those questions created a “mentality of ‘I’m a senior over you.’” P77 also said that high-turnover rates made relationships more level and fairer, suggesting that turnover also brings advantages to the workplace. P21 reported having her knowledge of residents dismissed by CX2s (i.e., officers who work directly with residents and have a caseload) who “had never worked in control posts” was his source of stress. P21 associated power centralization with a lack of trust, reporting that many individuals in a position of leadership had no knowledge of the reality on the ground nor trusted the intel officers had on residents:

They [CX2s] don’t actually know the inmates and what’s happening with the inmates when they’re in the office. They don’t see what’s going on. We talk about the inmates, sometimes we’re like, as CX1, we were talking about inmates, and we’re like: “That inmate [is] really needy. He’s [the inmate] always complaining about something.” And the CX2s are like: “No, we never had any issue with them.” So, I would say CX2 [is my source of stress] (P21).

Seniority also advanced a work ethos that required “newbies” to prove their worthiness before they were entitled to basic human respect. However, worthiness was not measured in merit and knowledge of policy or prison dynamics. Instead, worthiness had to do with the newer COs’ ability to mimic members from the established group and endure disrespect. For example, P87 revealed that senior officers refused to learn his name:

A lot of the old dogs don’t want to learn your name. Because you might not be here long enough. You either quit or you transfer. Until you’ve earned your name, it’s kind of like how they always say it, “when you earn your name” (P87).

P25’s co-workers never referred to her appropriately until she proved herself. Instead, she was referred to as “Susan’s cousin,” as her cousin “Susan” (alias) worked in the same prison. This kind of attitude, in addition to being demoralizing, could be experienced as oppressive. For instance, P25 suggested she never complained to her co-workers, keeping her “head down.”

I just got labeled as Susan’s cousin rather than as a person, and I think that was hard too. I try not to let it bother me. I just kinda keep my head down and do the job that’s required of me (P25).

### Management

Participants reporting management as their primary source of stress emphasized three stress triggers. These triggers are power centralization, communication issues, and unsupportive management. Each of these triggers provides insights to understand the meanings underpinning stress coming from management.

#### Power Centralization

Power centralization often resulted in unfeasible policies and protocols, which created opportunities for tension and arbitrariness in the workplace, leading to organizational injustice and compromised employee morale. P31 provided insights into how managers without front-line experience generated unrealistic orders, which COs were forced to execute despite deeming them incorrect and unsafe. Referring to “people in very high positions” within CSC” who have never “worked on the front lines” as unpragmatic, P31 said: “They imagine how something should be done rather than having practical hands-on experience; [they do not ask] “can it be done?” It’s more of an ‘it will be done!’”

P119 added that centralized decision-making processes jeopardize the safety of COs, residents, and prison security. P119 thought of centralization as the norm, not an exception, at her institution. In fact, dangerous decisions were so common at her institution that she reported not getting “stressed out anymore”; instead, she “just expected it”: “In the beginning, it [stress] was definitely from the calls that colleagues or management was making that you could see were not the smartest or the safest necessarily.”

Centralization created a chaotic work culture marked by arbitrariness and disregard for policy. P7 reported the following: “Half of the time they [residents] won’t even bother with us [COs]; they’ll just [do] all [they] want because they know they’ll [managers] override anything we [COs] say or do even if we’re following policy right.” P7 feared looking inconsistent in the eye of residents, which could compromise the CO-resident relationship: “If I’m trying to do something, management may reverse that, and then it makes us look bad.”

P10 also explored the impact of centralization on organizational justice. He reported several situations where correctional managers used mistakes arbitrarily disassociated from a critical incident to “penalize” or “try to fire” COs. Based on P10’s account, a CO half an hour late for his midnight round almost got fired unfairly. The management accused this CO of failing to prevent a resident’s death by suicide that happened 3 hr later after he had done his round and ensured that all residents were well in their cells. The CO perceived such an accusation as punishment for being late.

P104 attributed centralization to the “military aspect” of correctional work, equating correctional work to the armed forces. To this point, he said: “Like in the military, you’ll go by like a rank structure.” In his view, hierarchy resulted in those at the top often disregarding the needs and thoughts of inferior ranks:

You always go through the chain of command; that’s what they always said, right? I might be a little timid to go talk to a manager, knowing that they’re higher to me, they’re a higher rank, so they really don’t care about the issues that I have (P104).

P4, despite reporting management as “very understanding” (co-workers are his primary source of stress), shared how he avoids stress from management: “They’re [managers] easy to please if you do what they ask you to do and don’t talk back.” His comment suggests that blind obedience is a source of employee reward, corroborating P104’s comment on “rank structure.”

#### Communication Issues

Lack of instrumental communication in carrying out tasks and meeting expectations was another source of stress cited in connection with management. Communication issues were associated with ineffective communication channels and chaotic work routines, as well as compromised work performance, prison safety, and employee morale. Communication problems also affected COs’ ability to exercise their discretionary powers, an important skill in correctional work. P63 reported that residents at her institution often heard about information that was “pertinent” to correctional operations before COs, revealing a problem with communication channels:

Lack of communication and not knowing even how. A lot of times, it’s sad, but lots of times, the inmates know things before we know things, which is kind of sad. Something is going on with my inmate [in his case load], and nobody tells me. Nobody says anything to me that my inmates have done this. I’m not going to know, and some pertinent information, I really probably should know because I’m the one that’s dealing with lack of communication is a hard thing, I would say (P63).

P104 reported his main stress was being “bounced” between units because “all lines were full.” In addition, being constantly moved around residents prevented him from knowing the residents he cared for, which made him more vulnerable to manipulations and risky interactions. Detailing the situation, he said:

I could never really get used to one unit or population. It’s just not knowing where I’m going; I go to a spot where I’m not a hundred percent sure [about] the population or even the routine, and then that would cause a little bit of stress [was that] the inmates would see that, and it’s like: “Oh there’s this guy here, and we don’t know who he is, so let’s try to like manipulate him into doing something that we wouldn’t normally do. . .” (P104).

Officers in the control room are responsible for managing different flows of residents within the institution, which includes ensuring that rival groups do not meet up, which could lead to conflict and violence. Thus, lack of knowledge of the resident population limited P104’s job performance and ability to ensure staff and resident safety: “You wouldn’t know the population; being in the control bubble [control room] trying to put them [residents] away, [you] don’t know who’s who and stuff like that, so it got a little stressful there at times.”

Having to exercise discretionary powers amid unclarity posed a tremendous strain on participants. For instance, citing the Structured Intervention Unit (CSC, 2022b), Canada’s alternative for solitary confinement, as an example, P35 explained that unclear communication of the policy affected her stress level: “Like with those changes to segregation or whatever, officers are being told to use their discretion in certain situations, and then, when we do, we kind of get shit on anyway.” P35 reported that misinformation and lack of information led her to feel “pushed around” and “blamed” unfairly, associating a lack of instrumental communication with organizational injustice. Officers often experience such feelings as a form of disrespect (P35). After highlighting that correctional work is “not black and white,” P123 reported that the “gray areas” resulting from ineffective communication, those moments when “you don’*t* know exactly what to do,” were extremely stressful.

#### Unsupportive Management

Unsupportive management was another stress trigger in correctional work. Participants feared unsupportive management because it was often associated with distrust, disregard for evidence, arbitrary punishment, and organizational injustice. Officers believed that unsupportive managers posed a risk to staff and residents’ safety and security. P17 associated unsupportive management with a workplace marked by distrust and punishment-oriented scrutiny, as opposed to accountability, reliability, and fairness: “So, you know, management is always looking over your shoulder.” Thus, he expected to receive no support if he needed to use force against a resident. Instead, he expected to be “crucified”:

Probably management and the public [sources of stress] because inmates, you can deal with them; the violence you deal with it as it’s happening. But, if you shot somebody or something happens, then you’re probably going to be crucified by the public and management (P17).

P45 suggested that resident-on-CO assault at her institution went unpunished because of unsupportive managers. P45, discussing a situation in which she received death threats from a resident, reported the warden was “more concerned” about the individual’s well-being than hers. According to her, the warden checked on the resident placed in segregated housing because of the incident “every single day.” However, the warden never checked on her. Also, the warden dismissed the option to transfer the resident to another correctional facility. Instead, the warden opted to remove the resident from segregation back into the “general population” as soon as possible, making the CO concerned for her safety work. In a group meeting about the incident, P45 confronted the warden and complained about his unsupportive attitude:

I spoke up, and I was nervous, so, so nervous, and I was like: “Every single day for over 30 days you went down to segregation and asked this inmate how he was doing, not once have you asked me how I am” (P45).

She described feeling the warden’s disregard for her well-being caused her stress and prolonged strain: “No one deserves to have their life threatened coming to work. I get what I signed up for, but that’s [death threat] not part of the job.” P45 also suggested that unsupportive management and impunity can undermine the goal of correctional work. Paraphrasing what she told the warden, she told the interviewer: “I’m trying to help correct them [residents] and get them back into society. I have my life threatened, and you [the warden] want to keep him [the individual who threatened her] here? Why? That’s what I want to know? Why are you keeping him here?” (P45)

P45 was not the only participant discussing resident-on-CO assault going unpunished. P49 disapproved of management showing lenience toward residents who attack officers, feeling managers should temporarily curb the rights of residents who attack officers: “Supporting inmates when they do staff assaults and still letting them have visits and things of that nature. Like it wasn’t directly associated to me but clearly, if you’re within the area, it’s not gonna sit well with you.” P49’s comments revealed that leniency toward resident-on-CO assaults compromised the entire team (employee morale), not only the assaulted CO.

P106 reported that “intense oversight of management” associated with mistrust and lack of support aggravated his stress when dealing with residents. His managers dismissed the knowledge officers produced of residents as legitimate evidence, which often resulted in the officers being punished for relying on that knowledge to determine their course of action in case of incidents. To justify his concerns, P106 recounted an incident in which “a very caring” officer decided to take a “quite strong and unpredictable” resident down “with a body block” to stop them from assaulting another officer. As the individual was known to “laugh at” pepper spray, the CO responding to the incident took them down. In the fall, the prisoner “bumped his head on the floor and opened a small cut over his eye.” Although the CO’s response freed the CO from being victimized, the responding officer was punished (i.e., moved out of the post and considered for disciplinary actions). Although the responding officer was eventually cleared of any wrongdoing, the event affected P106 vicariously, leaving him insecure and in fear of doing his job. Like P106, P125 also suggested that managers did not trust the information and knowledge officers have of residents, which created a lot of anxiety among staff: “The way they [managers] decide to do some things. . . It seems like we tell them one thing [and] they [residents] tell them another. Then, it’s a battle between what’s actually going to happen.”

P90, an officer who had worked in Canada’s provincial correctional system, reported being surprised with what residents “get away with” in the federal system. P90 suggested that impunity also triggers stress: “It’s kind of surprising to see what they allow each other to get away with here! They’re just a little more tolerant, I guess, because they spend more time with these guys [residents]. They’re not on remand time, so it’s not just a couple of weeks, and they’re out.”

### Residents

Most participants indicating residents as their primary source of stress summarized their stress as coming from “the job,” viewing interactions with prisoners as the heart of correctional work. For instance, P2 said: “It’s the job; that is what I want to say. Yeah, dealing with inmates; that probably causes me the most stress.” P19 echoed P2: “Well just for one, your job is always going to be dealing with inmates. I mean that’s always going to be your primary focus. I guess while you’re at work and kind of where most of the stress comes from.” Uncertainty and unpredictability, demands amid short staffing, and role conflict were the main problems raised in connection with residents. It is noteworthy that only 8 out of the 13 participants who named residents as their source of stress provided a detailed rationale.

#### Uncertainty and Unpredictability

Participants often viewed residents as unpredictable. They reported the CO-resident interactions as marked by uncertainties and risks that could ultimately jeopardize the officers’ safety. P56 reported feeling stressed about having to “handle” residents:

Obviously, uh just dealing with them [residents]. . . Whether it be talking to them, dealing with them, or having to go put cuffs on them, on an inmate and handle them that way or whatever. It’s just you know, it’s kind of an unknown variable. It depends on how the people or how the population’s feeling that day (P56).

P56’s comments suggested perceiving and anticipating residents’ attitudes, which he portrayed as an “unknown variable,” was a core skill in correctional work. Knowledge of residents can potentially mitigate the stress resulting from the prison population’s uncertainty. Discussing the empirical consequences of navigating uncertain interactions, P107 reported that his safety and the safety of his co-workers depended on his ability to “pay attention”:

Ah, the uncertainty! There’re days it can be unpredictable. I shouldn’t get too complacent here. I mean you’re always on edge; a little part of you is always on edge because you never know when something going to go on. I mean it’s the uncertainty that can be stressful. Obviously having 600 inmates running around. . . I mean once again you also learn how to pay attention as long as you pay attention, I’d say this to anybody: “As long as you’re paying attention, you’re going to be okay” (P107).

#### Demands Amid Short Staffing

COs had to deal with numerous demands from residents amid short staffing, which difficulties to recruit employees and high turnover rates only aggravate. Many participants found extremely stressful due to pressure and even threat from residents. For instance, P30 mentioned that residents often intimidate COs:

A lot of them [residents] come in and expect something to be done and it has to be done right now. And then they start to threaten and you’re like OK, there’s a way we do things, I’ve got a bunch of other inmates to deal with and very demanding (P30).

Echoing P30, P54 said residents “constantly roared” at COs with ever-increasing demands: “Like always putting you down like do this for me and do that for me. I guess they just expect way more.” When P54 was asked to detail what else (i.e., “more”) residents expect from officers, P54 replied, “more than your job titles,” alluding to tasks beyond the responsibility of the post. For instance, according to P54’s example, residents, oblivious to how work is organized at the institution, asked COs working in the control room to fulfill demands that require leaving the post and going down to the “ranges.” Despite P54 describing residents as being “impatient,” she acknowledged that assisting residents could take some time because her unit had just a handful of COs for 70 residents, exposing the impact of understaffing on stress. P117, who also reported that residents’ demands make him stressed, suggesting that understaffing raises the risk of violence in correctional institutions:

I don’t know why they [residents] expect everything right away. This bubble has 4 units; the policy says we’re supposed to run it with one staff member in here. [However], if we ran it with one staff member we would have a riot on our hands every single day, so we put 2 staff members in here to lessen the workload and it’s still insane (P117).

#### Role Conflict

Balancing care and custody—the two distinctive and often contentious roles—within the correctional routine was also stressful. P38 reported “biting his tongue” and “picking his battle” while doing routine work. According to P38, depending on what went on, COs could only “roll their eyes,” or “brush it off the shoulder,” and “keep walking.” To “pick their battles,” COs relied on discretionary power. However, exercising discretion did not come without stress.

### Co-Workers, Management, and Residents: Comparative Perspectives

Participants reporting staff as their primary source of stress were often inquired into why they did not view residents as stressful. Most attributed staff stress to a mismatch between job expectation and job reality, including a discrepancy between the procedures taught at the correctional training academy and the reality of the job. Most participants joined the correctional career expecting interactions with residents to be stressful. However, they did not anticipate interactions with peers to be “so” toxic. For instance, P27 said: “I suspect you kind of know what to expect from the offender population but not necessarily what to expect from the guard population.” P119 said that he expected better behavior from fellow officers: “The inmates you expect them just to be the way they are like that’s not going to change. They’re here for a reason kind of thing, but I tend to hold co-workers to like a higher standard.” In the same fashion as most participants who named staff as their primary source of stress, P77 said:

Inmates never stress me out. It’s the people that I work with because I expect more from them than I would from the inmates because the inmates have their own. . . And I mean I can say that with the staff too, they have their own maybe crap going on, but I don’t know. The inmates I don’t expect—I don’t—that doesn’t burn me out (P77).

P31 discussed the “pushbacks” he received from “older officers” for trying to apply what he learned in correctional training: “That’s the most stressful thing. . . Inmates trying to assault me?! Shit! I expect that to happen! Yeah, I expect it!” P5, with 14 years of correctional experience in the correctional system, explained that receiving bad peer evaluations is inevitable in correctional work. Echoing P31, P5 suggested that a lot of the stress newer officers feel results from a disparity between what officers learn in training and the reality of correctional work, which is extremely concerning for COs and residents. In P5’s words, “new ones will come straight from Core [correctional training] and try to do what Core teaches you to do. . . [Core teachings] don’t always transfer into real life when you’re here.” Conversely, P5 also mentioned that management also contributes to increased stress on the job.

## Discussion and Conclusion

The current study openly inquired 44 COs from 12 federal correctional institutions in Canada about their stress sources after completing a year on the job. In a rare endeavor, the study gave COs a voice to name and explain their sources of stress instead of surveying them with a predetermined list of stressors and stress triggers. Thus, this study offered a window into the problems that recently hired COs experience on the job.

Key findings indicated staff as a significant source of stress in correctional work. Over two thirds of participants viewed staff (i.e., co-workers or managers) as their primary source of stress, while about a third viewed residents as their primary stressor. Overall, research findings converged with the broad correctional literature, which has indicated unsupportive support (Breen, 1986; Owen, 1988; Tewksbury & Higgins, 2006; Walters, 2022) and unsupportive management (Butler et al., 2019; Hughes & Zamble, 1993; Peeters & Buunk, 1995; Schaufeli & Peeters, 2000) as stressful. Like in most sectors, staff can make or break the correctional workplace. However, unlike the correctional literature, the current study provided insights into the meanings underpinning the stress coming from peers and managers.

Gossip and seniority were the most significant stress triggers associated with co-worker stress. Gossip is a human strategy to manage relationships, and it exists in every social group (Dunbar, 2004; John et al., 2019). However, the volatile nature of correctional work, often justified in prison violence, aggravates the effects of gossip and its consequences on COs’ well-being. Participants feared that being seen and talked about as a “burden” could affect their co-workers’ willingness to respond and help during critical incidents (e.g., resident-on-staff assault). In sum, gossip seems to compromise the CO’s perception of workplace safety. Widespread gossip led participants to experience anxiety, fear of ostracization, isolation, and stress. In addition, participants suggested that gossip is rooted in boredom, a long-standing characteristic of correctional work (Philliber, 1987). The correctional literature has identified boredom as a stress determinant (Dollard & Winefield, 1995; Hughes & Zamble, 1993), but it has left, until now, the relationship between boredom and gossip unexplored. Thus, the current article offers a pathway to further explorations involving boredom and gossip, especially in maximum security prisons, where COs tend to sit in the “bubble” for 12 or 16 hr, sometimes even longer.

Seniority is often discussed positively, as it tends to organizational commitment (Brown et al., 2019). However, research findings demonstrated that seniority also could fuel and legitimize an oppressive social order that subjected new hires, often referred to as “newbies,” to disrespect and humiliation until they proved trustworthy. New group members in any community undergo a transition phase that often includes gossip, stigmatization, and exclusion. This transition phase is part and parcel of the process through which “outsiders” prove their worthiness to become “established” (Elias & Scotson, 1994). However, in correctional services, proof of worthiness often involves blind obedience to hierarchy and a disregard for protocols taught at the correctional training program, which is extremely concerning. Training and protocols are the only guarantees of organizational justice, accountability, security, and safety in corrections. To be clear, blind obedience to hierarchy instead of protocols in a closed and coercive environment like corrections may open the door to resident and staff abuse and thus compromise the legitimacy of a correctional system. Despite its potentially harmful effects on correctional staff and residents, seniority has received limited attention from correctional scholars. The few studies that explored seniority limited their analyses to the impact of seniority on CO’s professional orientation, i.e., if seniority contributes to a punitive or a rehabilitative orientation (Farkas, 1999; Moon & Maxwell, 2004a; Toch & Klofas, 1982), leaving workplace abuse untouched.

Furthermore, findings about using seniority to acculturate new hires raise questions about how and to what extent hierarchy and chain of command (i.e., the paramilitary nature of corrections) can facilitate correctional services—another topic left unexplored by the correctional literature. Can paramilitary organizations grounded in authority foster a more humane correctional rationale? In an era where correctional services, like Canada’s federal corrections, officially refer to residents as “clients,” the paramilitary nature of such services must be analyzed for adequacy and called into question.

Stress triggers associated with management also deserve attention, starting with power centralization. Overall, findings corroborated existing studies presenting centralization as a stress correlate (Cheek & Miller, 1983; Lindquist & Whitehead, 1986; Saylor & Wright, 1992; Wright et al., 1997). Power centralization, like seniority, creates opportunities for an authoritarian organizational culture that disregards protocols and disciplined employees without considering their input and evidence. In other words, findings confirmed that power centralization, as the correctional literature indicates, can compromise organizational justice (Lambert et al., 2006). Power centralization can also lead to the development of unfeasible policies that strain the COs who must implement them. Overall, the organizational culture within correctional services seems to contrast with the culture of highly effective for-profit organizations drastically. Although correctional services use mistakes, negligence, and wrongdoings to punish individuals, treating them as “bad apples,” for-profit organizations tend to use mistakes, negligence, and wrongdoings to improve protocols, address structural problems, and advance more efficient work processes. Such a differential in the approach to mistakes, negligence, and wrongdoings results in a culture of fear at corrections and a culture of high productivity and teamwork in for-profit organizations.

Lack of effective communication was another stress trigger linked to management. The correctional literature has demonstrated that communication issues, often associated with power centralization, contribute to chaotic work environments where everyone does things differently, with the caveat that newer officers do not enjoy the same level of autonomy as their seniors (Lambert et al., 2006, 2010). Meanwhile, findings contributed to the literature by emphasizing another issue related to communication: lack of instrumental or effective communication compromises the COs’ ability to produce knowledge of the correctional population. Insufficient knowledge of the prison population directly affects the officer’s ability to make the right decisions to keep the prison, including residents and staff, secure and safe. Also, officers who do not make the right call during critical incidents risk disciplinary consequences. Thus, communication issues can challenge organizational justice (Lambert et al., 2006, 2007, 2019) and cost COs their careers.

The correctional literature has extensively explored the concept of unsupportive management. However, the literature has not really delved into the meanings of unsupportive. Findings revealed that unsupportive managers are those managers who disregard evidence, punish their subordinates arbitrarily, and promote organizational injustice, that is, individuals not deemed reliable or trustworthy or caring. Unsupportive management allows individuals, including staff and residents, who victimize COs or break prison rules to go unpunished, which is unfair to COs and the residents who follow the rules. In addition, managers who leave the victimization of employees unaddressed contribute to diminishing employee morale. Low morale resulting from resident-on-staff abuse and violence can foster a dangerous divisiveness between COs and residents. Managers with a loose understanding of organizational justice can also let staff-on-resident abuse and violence go unnoticed or unpunished. Overall, unsupportive managers facilitate a culture of impunity within correctional services. Finally, any factor contributing to a culture of impunity and arbitrarity within corrections deserves attention because such a culture can disregard, omit, or downplay violence involving staff and residents.

Participants indicating staff as a source of stress often highlighted that they joined the correctional career expecting residents to be a source of stress, not co-workers. Such a mismatch between job expectations and job realities may have heightened COs’ perceptions of co-workers as a source of stress. Studies focused on human resources have shown that discrepancies involving job expectations and realities can compromise job performance (Savery et al., 1983) and job commitment (Lambert, 2003; Lambert et al., 2006), in addition to generating stress.

Unique ambiguities and specificities mark the CO occupation, making the need to address stress in this line of work particularly important. First, COs are agents of care and coercion simultaneously. Second, the CO’s workplace is also someone’s home. Third, COs exercise authority over a population that is physically and mentally vulnerable. Because of such ambiguities and specificities, work conditions and conditions of incarceration fully overlap in this line of work. Thus, the goal of transforming the prison system into a fair, safe, and secure correctional environment for residents cannot be achieved without considering the COs’ occupational health and safety, including their sources of stress.

The current study includes advantages and limitations, mostly related to its sample’s profile. Recently hired COs offer a fresh view of correctional work and its problems. However, despite their seriousness, new hires challenge problems that have become normalized for correctional practitioners and correctional scholars. In doing so, newer officers allow correctional researchers to see the strange in the familiar (Mills, 1959). For instance, participants’ input on seniority allows for calling into question the paramilitary organization of correctional work and its effectiveness amid a growing movement to destigmatize residents and transform correctional services into a rehabilitation opportunity instead of a punishment regime. However, newer officers have limited contextual knowledge of the social dynamics that mark the correctional workplace, producing a partial interpretation of the reality underpinning occupational stress. Thus, it is necessary to consider that some of the underlying stress triggers, such as gossip and seniority, also may have a social function within correctional service.

Gossip represents a source of knowledge about prison life and a strategy for developing social bonding (Dunbar, 2004). The knowledge and social bonding that gossip produces contribute to the prison’s security and safety. The same is true about seniority. Seniority fosters employee commitment to a lifelong career (Gunawan & Marzilli, 2022), which enables institutional memory; senior employees are repositories of valuable unwritten information about work practices, which can benefit new hires (Kleinman et al., 2002).

The current study provides limited opportunities for policy recommendations due to its sample size and exploratory nature. However, findings can illuminate, yet partially, official portrayals of Canadian federal correctional institutions as toxic workplaces (The Correctional Investigator Canada, 2019; CSC, 2020) and confirm that prisons in Canada deserve immediate attention as a workplace. Future research should continue to explore the reasons underpinning staff as a source of stress. A comprehensive understanding of the social dynamics behind stress coming co-workers and managers could provide correctional practitioners and stakeholders contextual information to improve correctional workplaces, positively affecting the already compromised COs’ well-being (Regehr et al., 2021). Improving prisons as a workplace would also positively affect the quality of services provided to residents. The paramilitary nature of correctional services and its intended and unintended consequences on correctional work also should be examined. Military discipline is associated with unquestionable obedience to authority (Weber, 1978), offering a fertile ground for disrespectful and abusive behaviors. Re-thinking corrections as a paramilitary organization and developing alternatives may help address the “culture of impunity and mistreatment” (The Correctional Investigator Canada, 2019), especially involving staff-on-staff violence, that has tainted the reputation of Canada’s federal correctional services.
